# Impacted wooden toothpick in the stomach

**DOI:** 10.1002/ccr3.1903

**Published:** 2018-11-05

**Authors:** Shinsuke Yahata, Momoka Kamada, Tsuneaki Kenzaka

**Affiliations:** ^1^ Division of Community Medicine and Medical Education Kobe University Graduate School of Medicine Kobe Japan; ^2^ Department of General Medicine Toyooka Public Hospital Toyooka Japan; ^3^ Division of Community Medicine and Career Development Kobe University Graduate School of Medicine Kobe Japan

**Keywords:** epigastric pain, gastric foreign body, toothpick

## Abstract

Foreign body ingestion should be considered when evaluating acute onset epigastric pain, even if patients have no recollection of foreign body ingestion and suspicious conditions or habits, especially in the regions where toothpicks are used on a daily basis.

## 1 CASE REPORT

**Figure 1:**
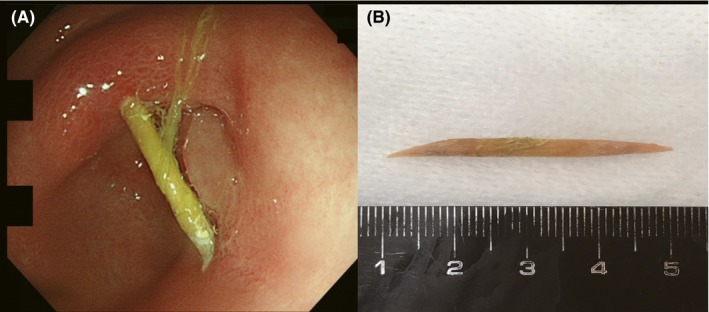
A, Esophagogastroduodenoscopy showing a linear foreign material stuck in the lesser curvature of the gastric antrum. B, The broken wooden toothpick which was approximately 4 cm long

A 28‐year‐old woman presented with epigastric pain. She had eaten sushi 1 day prior and developed intermittent epigastric pain after approximately 15 minutes. She did not have nausea or diarrhea. She was in the early pregnancy condition, and there was no abnormality during pregnancy. She had mild epigastric tenderness and no rebound tenderness or guarding. Her laboratory, electrocardiogram, and abdominal ultrasonography results were normal. She did not undergo radiography and computed tomography because of pregnancy. We suspected anisakiasis initially; therefore, esophagogastroduodenoscopy was performed. Her gastric mucosa was nonatrophic, and there was a linear foreign material stuck in the lesser curvature of the antrum (Figure 1A). We grasped the end of the material, which was hard, with alligator forceps and took it out carefully. There were no bleeding and signs of perforation; then, we clipped the wound in order to be certain that bleeding and perforation will not occur. Subsequently, we removed the foreign body with a sharp tip pointing toward the caudal side, without using any device to protect the esophagus, and we determined that the material was a broken wooden toothpick, approximately 4 cm in length (Figure 1B). Although we recommended that the patient to be admitted to our hospital, she did not agree; therefore, we carefully conducted outpatient follow‐up. We instructed her to fast for 1 day and eat fluid diet from the second day onwards. Three days later, she returned to our hospital with no symptoms, she was subsequently permitted to eat regular meals.

## 2 QUESTION

Why not did she remember swallowing the toothpick?

## 3 ANSWER

Numerous cases of toothpick ingestion have been reported.^1^, ^2^ Typical clinical complaints of toothpick ingestion are digestive symptoms, such as abdominal pain, nausea, diarrhea, and fever.^1^ Toothpick ingestion was detected on endoscopy and/or computed tomography and ultrasonography; and endoscopy had high sensitivity (72.1%).^1^ Major complications of toothpick ingestion were digestive perforation and migration into adjacent organs. Fortunately, our case had good prognosis. Diagnosis and treatment should be done cautiously if foreign body ingestion is suspected. There are other known several risk factors for foreign body ingestion, such as alcohol consumption, eating food that contains toothpicks, habitual chewing of toothpicks, and dental plates,^1^ but this case did not have any of those conditions or habits. Furthermore, the majority of patients (54%‐88%) do not realize that they have ingested a foreign body.^1^, ^2^ Our results suggest that foreign body ingestion should be considered when evaluating acute onset epigastric pain, although the patient may have no memory of foreign body ingestion, and does not have any suspicious conditions or habits. This is especially relevant in regions where toothpicks are used on a daily basis.

## INFORMED CONSENT

Informed consent has been obtained for the publication of this clinical image from the patient.

## CONFLICT OF INTEREST

None of the authors have any financial interests to disclose, nor do they have any conflict of interests to declare.

## AUTHOR CONTRIBUTION

SY and TK: involved in conception and design. SY and MK: drafted the article. TK: reviewed the manuscript for critically important intellectual content. SY, MK, and TK: involved in final approval of the article.
